# Longitudinal outcomes of final kissing balloon inflation in coronary bifurcation lesions treated with a single stent

**DOI:** 10.3389/fcvm.2023.1290024

**Published:** 2023-11-30

**Authors:** Lefan He, John F. Robb, Pablo Martinez-Camblor, Bruce W. Andrus, Lily J. Greene, Prajesh Gongal, Dhruthi S. Reddy, James T. DeVries

**Affiliations:** ^1^ Department of Cardiology, University of Rochester Medical Center, Rochester, NY, United States; ^2^ Heart and Vascular Center, Dartmouth-Hitchcock Medical Center, Lebanon, NH, United States; ^3^ Homer Stryker School of Medicine, Western Michigan University Homer Stryker School of Medicine, Kalamazoo, MI, United States

**Keywords:** PCI, bifurcation, coronary, stent, inflation

## Abstract

**Background:**

Final kissing balloon inflation (FKBI) is a percutaneous coronary intervention (PCI) technique that is considered mandatory to improve outcomes in two-stent strategies, but its use in single-stent bifurcation PCI remains controversial.

**Methods:**

In this retrospective cohort study, we identified patients with coronary bifurcation lesions treated with one stent from January 2012 to March 2021 at a single academic medical center. Incidence rates per 1,000 patient-years (IR1000) were calculated for the outcomes of all-cause mortality, myocardial infarction (MI), stent thrombosis (ST), target lesion revascularization (TLR), coronary artery bypass graft (CABG), and cardiac readmission between patients who received FKBI and those who did not over a median follow up of 2.3 years. Studied outcomes were adjusted for all baseline clinical and procedural characteristics.

**Results:**

This study included 893 consecutive patients of which 256 received FKBI and 637 did not. The IR1000 for MI were 51.1 and 27.6 for patients who received FKBI and patients who did not, respectively (adjusted HR = 2.44, *p* = 0.001). The IR1000 for death were 31.2 and 52.3 for patients who received FKBI and patients who did not, respectively (adjusted HR = 0.68, *p *= 0.141). The incidence rates of ST, TLR, CABG, and cardiac readmissions were similar between patients who received FKBI and those who did not.

**Conclusions:**

These results suggest that performing FKBI in a one-stent technique was associated with higher rates of myocardial infarction, particularly in the first 6 months, and no difference in death, ST, TLR, CABG, and cardiac readmission rates.

## Introduction

Coronary bifurcation lesion interventions represent twenty percent of all percutaneous coronary interventions (PCI) and are associated with increased rates of mortality, stent thrombosis (ST), and target lesion revascularization (TLR) (1). Mechanistically, interleukin (IL) 6, IL-1β, and NLRP-3 inflammasome are crucial mediations that have all been associated with the development and progression of coronary artery disease (2–4). Recently, the CANTOS randomized clinical trial found that patients with somatic variants in *TET2* who were given canakinumab, an anti-IL-1β antibody, demonstrated reduced risk for major adverse cardiac events (MACE) (5). These studies support the rationale of targeted anti-inflammatory treatment in high-risk patients.

Current European Bifurcation Club (EBC) guidelines recommend a one-stent strategy for the vast majority of bifurcation lesions, but an up-front two-stent strategy is recommended in the setting of complex bifurcation lesions with large and diseased side branches (SB) (6). Up-front, single-stent strategies have been shown to produce similar or improved clinical outcomes as some dedicated two-stent approaches, with the additional benefit of decreased procedure time, radiation exposure, and cost (7–12).

Final kissing balloon inflation (FKBI) is a PCI technique considered mandatory for any two-stent approach as this has been associated with improved outcomes (6, 13). However, use of FKBI for one-stent techniques remains controversial (14). FKBI may improve clinical outcomes in PCI by reducing “jailed” and floating struts after stent deployment in retrospective and *in-vitro* studies (15, 16). However, other studies have shown the potential for kissing balloon inflation to cause overexpansion of the proximal segment of the stent in the main vessel, leading to elliptic deformations, impaired antiproliferative effects, reduced drug delivery, and increased restenosis risk (17, 18). Thus, the translation of improved side branch stent geometry to better clinical outcomes has been mixed.

Several large randomized controlled trials and meta-analyses have evaluated the benefits of FKBI in one-stent techniques and have demonstrated no difference in outcomes between patients who received FKBI and those who did not (19–22). However, a number of retrospective studies have found conflicting results, with some studies demonstrating higher rates of MACE associated with FKBI while others have found lower rates of MACE as well as MI and death associated with FKBI (12, 23, 24). Given this ambiguity, the present study examined the long-term outcomes of final kissing balloon inflation in patients following bifurcation PCI with a one-stent strategy at a single, tertiary care institution.

## Methods

### Data collection

The Dartmouth Dynamic Registry, a consecutive, prospectively collected electronic database of all cardiac catheterizations performed at our institution, was queried from January 2012 to March 2021. The query searched for all patients who were found to have at least one coronary bifurcation lesion that underwent PCI within the specified timeframe. The initial catheterization involving the bifurcation lesion was labeled the index case and all subsequent catheterizations were also evaluated. A bifurcation lesion was defined as a narrowing of a major epicardial artery occurring adjacent to or involving a side branch artery greater than or equal to 2 mm (visually estimated) in diameter. Baseline clinical characteristics and procedural data were collected. Variables collected included the indication for catheterization, location of the bifurcation lesion, the type of intervention performed, whether FKBI was conducted, case duration, fluoroscopy time, and volume of contrast administered. The locations of bifurcation lesions were grouped into one of four territories: left main coronary artery (LM), left anterior descending coronary artery (LAD), left circumflex coronary artery (LCx), and right coronary artery (RCA). Stent thrombosis (ST) was defined by the Academic Research Consortium classification (25). All cases of ST were confirmed by angiography and were considered definite ST. Target lesion revascularization (TLR) was defined as repeat PCI at the original lesion location. While ST could be classified under TLR, this study separated ST and TLR into separate outcomes. Procedural MI's were defined as occurring within 48 h of PCI. Patients with multiple bifurcation lesions in which at least one of the lesions involved a two-stent technique were excluded. In addition, patients with multiple bifurcation lesions were classified under the FKBI group if the patient was found to have at least one bifurcation lesion treated with FKBI. Patients with a history of CABG prior to their index bifurcation case were excluded due to non-native coronary anatomy. Lastly, patients with bifurcation lesions that were not *de novo* lesions were excluded. This removed lesions that had received prior PCI to either branch in the bifurcation lesion.

The institution's electronic medical record was also queried from January 2012 through July 2021 to evaluate for subsequent cardiac readmissions, myocardial infarctions, coronary artery bypass grafting (CABG), or death that occurred after the initial bifurcation PCI. Cardiac readmission was defined as any hospitalization for a primary diagnosis involving a cardiac pathology. Any subsequent myocardial infarction and CABG that occurred after PCI were determined through diagnosis codes. If a patient did not have a date of death, the patient's last encounter with our healthcare system was then noted as the last known date patient was alive.

### Statistical analysis

Continuous variables were summarized as mean ± standard deviation. Relative and absolute frequencies were reported for categorical variables. For group comparisons, we used robust Welch *T*-test or Chi-square test as appropriate. Incidence rates per 1,000 patient-years summarized the time-dependent outcomes. Naïve bootstrap method was used for computing both 95% confidence intervals and *p*-values for contrasting the equality among independent groups. Propensity score weighted Kaplan–Meier estimator was used for approximating the cumulative distribution functions of time-to-death and time-to-MI (26). Hazard ratios (and their respective 95% confidence intervals) from unadjusted and adjusted proportional hazard Cox regression models were used for summarizing the different behavior of the FKBI and no FKBI groups in the different time-to-event outcomes. Adjusted models included all listed baseline and procedural characteristics such as age, gender, hypertension, hyperlipidemia, diabetes, smoking status, prior PCI, left ventricular ejection fraction, stroke, dialysis dependence, case duration, fluoroscopy time, and indications for PCI. Two-sided *p*-values were reported and those below 0.05 considered statistically significant. Statistical environment R (www.r-project.org) was used for the statistical analyses.

## Results

**Figure 1:**
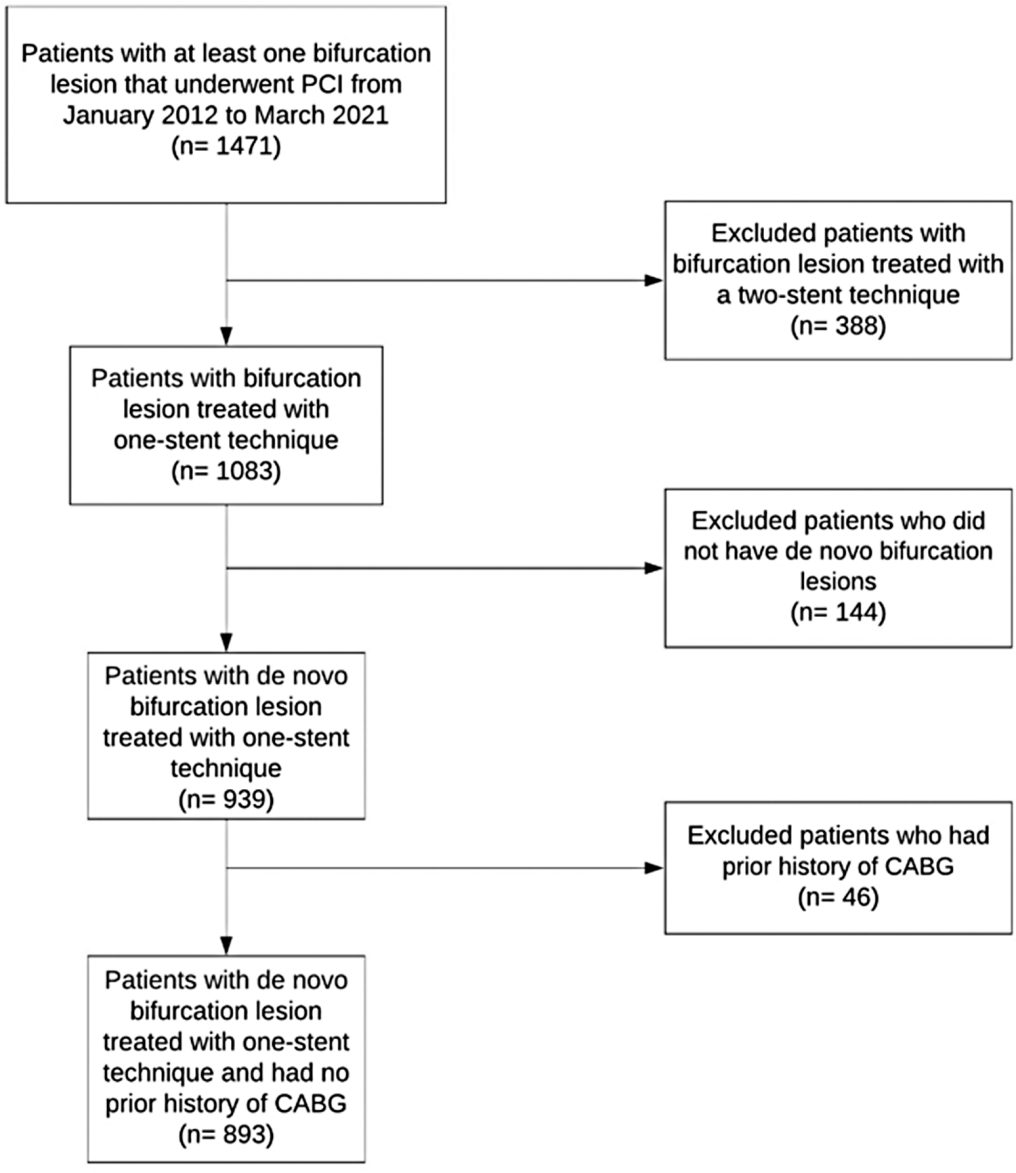
Study population flowchart.

We identified 1,471 patients with at least one bifurcation lesion that underwent PCI from January 2012 to March 2021. See Figure 1 for the study population flowchart. Of these 1,471 patients, 388 patients were excluded due to having a bifurcation lesion that was treated with a two-stent technique. Another 144 patients did not have *de novo* bifurcation lesions and were also excluded. Finally, another 46 patients were excluded due to a prior history of CABG. A total of 893 patients were identified with a *de novo* bifurcation lesion treated with a one-stent technique during cardiac catheterization from January 2012 to March 2021 and were included in this study (256 patients received FKBI and 637 did not receive FKBI).

The baseline characteristics are shown in Table 1. The median follow up was 2.3 years with a total of 2,507.2 patient-years. The mean age was 65.5 ± 11.6 years and 69.4% (442) were male. Diabetes mellitus was present in 31.3% (194), and hypertension was present in 71.6% (446). Patients who did not receive final kissing balloon inflation were on average 2 years older than those who received FKBI (*p* = 0.018). Otherwise, there were no statistically significant differences in baseline characteristics between study groups.

**Table 1:** Baseline characteristics.

	Final kissing balloon inflation not performed (*n* = 637)	Final kissing balloon inflation performed (*n* = 256)	*p*-value
Age, years	66.1 ± 11.9	64.1 ± 10.8	0.018
Male gender (%)	442 (69.4)	174 (68.0)	0.738
Hypertension (%)	446 (71.6)	177 (70.5)	0.815
Hyperlipidemia (%)	430 (72.3)	153 (66.0)	0.088
Prior tobacco use (%)	327 (52.6)	137 (55.2)	0.524
Diabetes (%)	194 (31.3)	76 (31.2)	1.000
Prior PCI (%)	118 (18.8)	47 (19.2)	0.962
LVEF < 35% (%)	37 (6.4)	17 (7.3)	0.770
Prior stroke (%)	23 (3.8)	9 (3.8)	1.000
Dialysis dependent (%)	6 (1.0)	6 (2.6)	0.159

Table 2 summarizes the procedural characteristics of our study population by patient case. The average total case time in patients who received FKBI was 6.1 min longer than in patients who did not receive FKBI (*p* = 0.024). The average fluoroscopy time in patients who received FKBI was 1.5 min longer than in patients who did not receive FKBI (*p* = 0.037). Patients who received FKBI also received on average 32.5 ml more contrast compared to patients who did not receive FKBI (*p* < 0.001). Lastly, patients who received FKBI were more likely to have two or more bifurcation lesions found on cardiac catheterization compared to those who did not receive FKBI (*p* < 0.001). Table 3 summarizes the coronary artery distribution of bifurcation lesions. The majority of lesions were located in the LAD followed by the LCx, LM, and RCA in descending order of prevalence.

**Table 2:** Procedural characteristics by case.

	Final kissing balloon inflation not performed (*n* = 637)	Final kissing balloon inflation performed (*n* = 256)	*p*-value
Case duration, mean ± SD (minutes)	98.9 ± 40.4	105.0 ± 34.7	0.024
Fluoroscopy time, mean ± SD (minutes)	21.3 ± 11.8	22.8 ± 9.2	0.037
Contrast used, mean ± SD (ml)	186.9 ± 78.7	219.4 ± 80.6	<0.001
Indication for PCI			
Asymptomatic CAD (%)	10 (1.6)	2 (0.8)	0.546
Stable angina (%)	107 (16.8)	41 (16.0)	0.854
Unstable angina (%)	106 (16.6)	45 (17.6)	0.811
Atypical chest pain (%)	5 (0.8)	2 (0.8)	1.000
Treatment for MI (%)	385 (60.4)	159 (62.1)	0.699
Post infarct ischemia (%)	6 (0.9)	1 (0.4)	0.671
Cardiogenic shock (%)	7 (1.1)	1 (0.4)	0.533
Other indication (%)	16 (2.5)	6 (2.3)	1.000

**Table 3:** Coronary artery location of individual bifurcation lesions (*n* = 970 lesions).

	Final kissing balloon inflation not performed (*n* = 679)	Final kissing balloon inflation performed (*n* = 291)	Percent receiving FKBI
Coronary lesion			
LAD (*n* = 570)	404	166	29.1% (166/570)
LCx (*n* = 230)	146	84	36.5% (84/230)
LM (*n* = 124)	98	26	21.0% (26/124)
RCA (*n* = 46)	31	15	32.6% (15/46)

The unadjusted clinical outcomes in patients who received FKBI and those who did not are shown in Table 4. Outcomes are reported as incidence rates per 1,000 patient-years (IR1000). The IR1000 for all-cause mortality was 31.2 and 52.3 for patients who received FKBI and patients who did not receive FKBI, respectively (*p* = 0.006). The IR1000 for MI was 51.1 and 27.6 for patients who received FKBI and for patients who did not, respectively (*p* = 0.015). The IR1000 for ST were 5.3 and 5.8 in patients who received FKBI and in patients who did not, respectively (*p* = 0.872). The IR1000 for TLR were 21.1 and 19.2 in patients who received FKBI and those who did not, respectively (*p* = 0.773). The IR1000 for cardiac readmissions was 122.4 and 157.0 for patients who received FKBI and those who did not, respectively and this was found to be not significant (*p* = 0.067). There was no statistically significant difference in incidence rates of CABG between those who received FKBI and those who did not.

**Table 4:** Unadjusted clinical outcomes^a^.

	Final kissing balloon inflation not performed (*n* = 637)	Final kissing balloon inflation performed (*n* = 256)	*p*-value
All-cause mortality	52.3 (41.9–63.9)	31.2 (20.2–44.0)	0.006
Myocardial infarction	27.6 (19.6–37.1)	51.1 (33.9–71.2)	0.015
Stent thrombosis	5.8 (1.7–10.7)	5.3 (0.1–11.9)	0.872
Target lesion revascularization	19.2 (11.6–27.7)	21.1 (10.1–34.9)	0.773
CABG	7.4 (3.4–12.2)	4.2 (0.1–9.8)	0.292
Cardiac readmission	157.0 (133.5–183.9)	122.4 (93.2–158.7)	0.067

^a^ Outcomes are reported as incidence rates per 1,000 patient-years with 95% CI in parentheses.

Table 5 shows the unadjusted and adjusted hazard ratios for the different studied clinical outcomes. The model adjusted for all baseline and procedural characteristics listed in Tables 1, 2. The hazard ratio (HR) for myocardial infarction remained statistically significant in both the unadjusted (2.11, 95% CI 1.35–3.30) and adjusted models (2.44, 95% CI 1.41–4.24). The HR for all-cause mortality was statistically significant in the unadjusted model (HR = 0.60, 95% CI 0.38–0.93), the significance was lost in the adjusted model (HR = 0.68, 95% CI 0.41–1.14). The remaining outcomes of ST, TLR, CABG, and cardiac readmissions remained similar between groups after using an adjusted model.

**Table 5:** Clinical outcomes using adjusted models^a^.

	Unadjusted		Adjusted	
	HR (95% CI)	*p*-value	HR (95% CI)	*p*-value
All-cause mortality	0.60 (0.38–0.93)	0.021	0.68 (0.41–1.14)	0.141
Myocardial infarction	2.11 (1.35–3.30)	0.001	2.44 (1.41–4.24)	0.001
Stent thrombosis	1.07 (0.28–4.13)	0.925	1.21 (0.25–5.80)	0.806
Target lesion revascularization	1.21 (0.60–2.43)	0.600	1.19 (0.53–2.68)	0.671
CABG	0.60 (0.17–2.16)	0.433	0.56 (0.14–2.24)	0.412
Cardiac readmission	0.88 (0.67–1.15)	0.340	0.83 (0.60–1.14)	0.254

^a^ Adjusted model outcomes were adjusted for all baseline and procedural characteristics.

**Figure 2:**
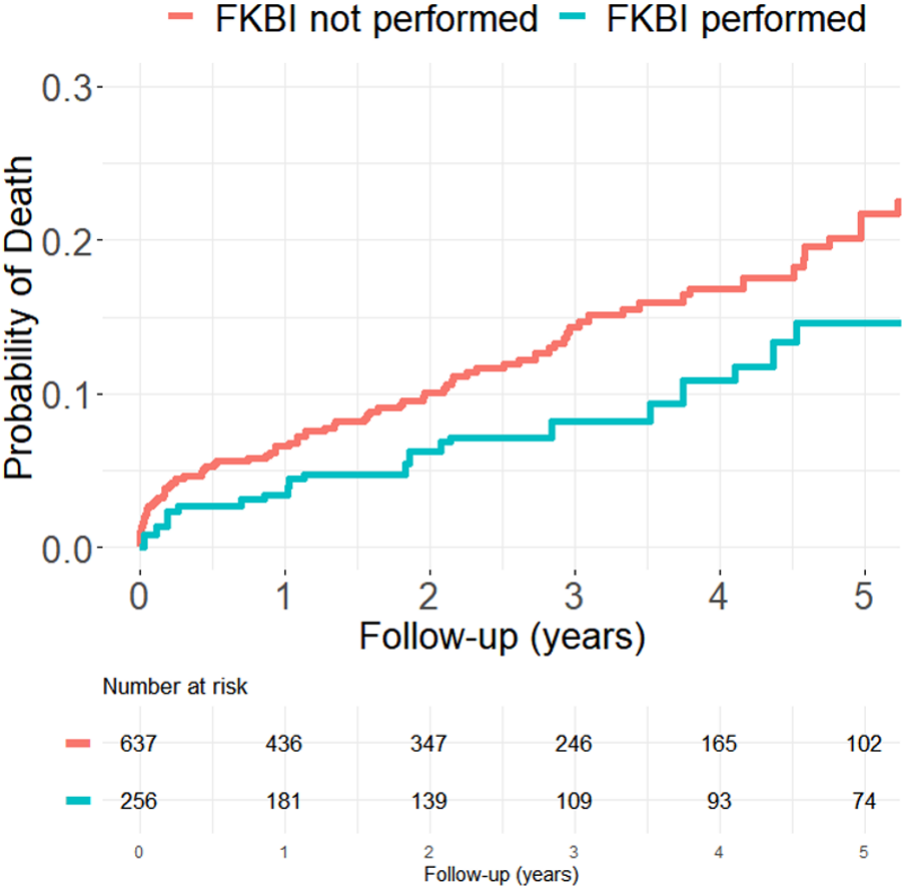
Propensity score weighted Kaplan–Meier estimation for all-cause mortality.

**Figure 3:**
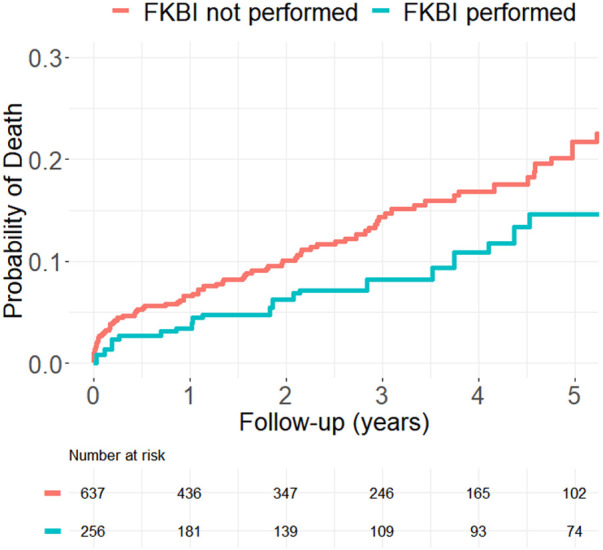
Propensity score weighted Kaplan–Meier estimation for myocardial infarction.

Figures 2, 3 show the propensity score weighted Kaplan-Meier estimations for the cumulative distribution function of the time-to-death and time-to-MI for both patients who received FKBI and those who did not. Patients who received FKBI experienced higher rates of MI mainly during the first six months. In the group that received FKBI, 42 MI's occurred within the first 6 months following PCI of which 3 of these 42 events were procedural MI's (occurring within 48 h of PCI). In the group that did not receive FKBI, 33 MI's occurred within the first 6 months following PCI of which 4 of these 33 events were procedural MI's. After these first six months, however, the difference in rates between groups remained relatively constant. Patients who received FKBI also appear to experience lower mortality rates compared to patients who did not receive FKBI, but the difference between groups was not statistically significant in the adjusted model.

## Discussion

This retrospective cohort study evaluated the longitudinal outcomes of FKBI in patients who received a one-stent strategy in the treatment of coronary bifurcation lesions. We found that FKBI was associated with higher rates of myocardial infarction when performed in a one-stent strategy, with the additional “cost” of longer case duration, longer fluoroscopy times, and larger volumes of contrast. Incidence rates of ST, TLR, CABG, and cardiac readmissions were similar between patients who did and did not receive FKBI. Lastly, there were no differences in all-cause mortality between groups after adjustment for baseline clinical and procedural characteristics.

The literature on FKBI in one-stent strategies remains controversial. While some randomized controlled trials, including Nordic-Baltic Bifurcation Study III and CORPAL, demonstrate no difference in MACE, MI, death, and TLR, these studies all have limited follow up to only one year (19, 20). Observational studies, which are inherently less rigorous compared to randomized controlled trials, have had longer follow up and demonstrated differences in outcomes between groups beyond one year. The sub-analysis of the TAXUS-PMS study, for example, had 3 years of follow up and found higher rates of MACE driven primarily by higher rates of TLR and target vessel revascularization (TVR) in the FKBI group compared to the group that did not receive FKBI (23). The COBIS II retrospective study also had a follow up period of 3 years but reported a decrease in MACE associated with the group that received FKBI (24). Notably, the difference in the rates of myocardial infarction between groups in this study occurred mostly within the first 6 months following PCI.

One possible explanation for the differences seen in clinical outcomes amongst various studies may be the indications for cardiac catheterization in the study population. Patients with non-ST-elevation acute coronary syndrome (NSTE-ACS, defined as NSTEMI and unstable angina) who undergo bifurcation PCI have been shown to have higher rates of MACE at 2 years follow up compared to patients with stable angina (27). NSTE-ACS itself was found to have borderline significance as an independent predictor of MACE in the present study (*p* = 0.06). Randomized controlled trials in the literature often enroll a majority of patients with stable angina (19). Similarly, many of the observational study populations had varying percentages of patients with stable angina, unstable angina, and MI, but with a low overall prevalence of MI as the indication for cardiac catheterization (15, 17, 24, 28). There has been only one retrospective study evaluating outcomes of FKBI specifically in patients presenting with acute coronary syndrome, which found FKBI was associated with lower rates of MACE, MI, and death (29). The majority of patients in the present study (60.9%) underwent PCI for the treatment of MI. Supplementary Tables S1, S2 presents the subgroup analysis of patients who underwent cardiac catheterization specifically for treatment of MI. Similar to the outcomes from the general study population, the clinical outcomes of this subgroup demonstrated higher rates of myocardial infarction without a statistically significant difference in mortality in patients who received FKBI. The heterogeneity in study populations and outcomes across the literature highlights the need for further studies differentiating patients undergoing bifurcation PCI for stable angina versus ACS.

In our cohort, the most common event was cardiac readmission with an incidence greater than 100 per 1,000 patient-years. Death and MI followed this with incidence rates per 1,000 patient-years ranging from 25 to 50 across the two groups. ST and CABG were both relatively uncommon with incidence rates below 10 per 1,000 patient-years. Incidence rate of death in patients who received FKBI (31.2) was almost half the rate in patients who did not receive FKBI (52.3) with a hazard ratio of 0.60. This difference dilutes in the adjusted models with a HR of 0.68 and the difference between groups also became statistically insignificant (*p* = 0.141). In contrast, the incidence rates of myocardial infarction in patients who received FKBI (51.1) were nearly double that of the patients who did not receive FKBI (27.6). Additionally, the adjusted HR (2.44) for death was slightly larger than the unadjusted (2.11) and the difference remained highly statistically significant after adjustment (*p* = 0.001). This highlights the increased risk of MI in patients who receive FKBI. Furthermore, the propensity score weighted Kaplan–Meier estimations show that the higher rates of MI seen in patients who receive FKBI mostly occurs within the first 6 months. There were 42 MI's that occurred within the first 6 months following PCI in the group that received FKBI and, of these 42 MI's, 3 (7.1%) of these events occurred within the first 48 h. In the group that did not receive FKBI, there were 33 MI's in the first 6 months of which 4 (12.1%) occurred within the first 48 h. Given the lower number of procedural MI's, this may suggest that the interventionalist chose to perform FKBI out of principle rather than out of necessity, such as in the event of side branch compromise leading to a procedural MI. After these first 6 months, the rates of MI between groups largely do not change and the curves remain parallel. One possible explanation for the higher rates of MI may be due to greater asymmetric stent expansion associated with FKBI when performed as part of a one-stent strategy (15). This asymmetric stent expansion has been shown to be an important determinant for thrombus formation (17). It is also possible that the side branch angioplasty site had recoil or restenosis from balloon trauma and the timing of the MI's would support this potential. Lastly, the rates of CABG in patients who received FKBI (4.2) was also much lower than in those who did not receive FKBI (7.4), with unadjusted and adjusted HR of 0.60 and 0.56, respectively. Because of the low number of CABG events, this difference between the groups was not significant. Rates of cardiac readmission, ST, and TLR were similar between groups and were also not found to be statistically different.

Many of the randomized controlled trials utilize MACE as an outcome, and few found differences in the individual outcome components of MACE (19, 20). However, in the case of COBIS II, lower rates of MACE with FKBI were driven mostly by lower rates of TLR (24). In the sub-analysis of TAXUS-PMS, higher rates of MACE associated with FKBI were driven by increased rates of TLR and TVR (23). The current study represents a population of patients severed by a rural, tertiary care academic medical center with a large hospital catchment area and a patient population that primarily receives all of their care at this institution as it is the only PCI hospital in the region. As such, we had more confidence in assessing individual outcomes over the time period studied.

One area this study did not investigate were outcomes associated specifically with left main bifurcations owing to the small number of LM lesions (*n* = 124). Randomized controlled trials have not been conducted evaluating FKBI exclusively in this patient population yet. Several observational studies have evaluated FKBI in one-stent strategies amongst patients with LM bifurcations and these studies have not found differences in the primary outcome of MACE (30, 31). Thus, there remains the need for randomized controlled trials evaluating FKBI in LM specific populations.

This study has several limitations. First, this is a retrospective study with all the inherent limitations to this design. Second, this study evaluated registry data from a single center. Third, this study evaluated patients who only received one-stent strategies and does not specifically evaluate provisional stenting, as the reason for choosing a one-stent strategy could not be ascertained. Thus, some patients who ultimately received two stents may have initially been planned as provisional stenting and were thus excluded from this study. Fourth, this study cannot determine why FKBI was or was not performed in each individual case. Having this information would have helped distinguish cases where FKBI was performed out of principle versus due to a compromised side branch. Fifth, the study did not have access to cause of death, and any deaths that occurred outside our electronic medical record would not be captured. Sixth, Patients who developed a subsequent MI after the index case were identified by diagnosis codes through our electronic medical record and, unfortunately, it is thus not known if the subsequent MI involved the original target lesion or occurred at a different site. Lastly, our dataset does not include whether intracoronary imaging was used during cardiac catheterization.

In this observational study, FKBI performed in bifurcation lesions treated with a one-stent strategy was associated with higher rates of myocardial infarction, particularly within the first 6 months (though rates of procedural MI were similar between groups). FKBI was also associated with longer procedure times, longer fluoroscopy times, and larger volumes of contrast utilization. There were no statistically significant differences in all-cause mortality after adjustment in patients who received FKBI and those who did not. Based on these findings, performing de facto FKBI in one-stent bifurcation strategies cannot be recommended. Larger-scale, prospective randomized trials with longer follow up periods are needed to further evaluate these findings.

## Data Availability

The data analyzed in this study is subject to the following licenses/restrictions: Please contact our IRB for access to dataset. Requests to access these datasets should be directed to DHMC IRB at irb@hitchcock.org.
