# Supplementary material for: Longitudinal outcomes of final kissing balloon inflation in coronary bifurcation lesions treated with a single stent
Source: Front Cardiovasc Med. 2023 Nov 30;10:1290024. doi: 10.3389/fcvm.2023.1290024 (PMC10720711; doi:10.3389/fcvm.2023.1290024)
Supplement: Supplementary file 1 [file Table1.docx]

Supplemental Tables 1 and 2 present the clinical outcomes of patients with coronary bifurcation lesions who underwent cardiac catheterization specifically for the indication of treatment of MI.

Supplemental Table 1. Unadjusted Clinical Outcomes of Treatment of MI Subgroup*

|  | Final Kissing Balloon Inflation Not Performed  (n = 385) | Final Kissing Balloon Inflation Performed  (n = 159) | *p*-value |
| --- | --- | --- | --- |
| All-cause mortality | 61.6 (46.3 to 79.5) | 37.8 (21.7 to 57.0) | 0.0351 |
| Myocardial infarction | 45.4 (30.8 to 63.2) | 90.0 (57.7 to 132.8) | <0.0001 |
| Stent thrombosis | 1.51 (0.00 to 5.28) | 7.22 (0.00 to 19.3) | 0.1204 |
| Target lesion revascularization | 19.7 (9.83 to 31.4) | 18.1 (3.62 to 37.6) | 0.8567 |
| CABG | 7.76 (2.44 to 14.7) | 0 | ---- |
| Cardiac readmission | 137.6 (108.8 to 171.7) | 114.9 (79.3 to 161.0) | 0.3312 |

*Outcomes are reported as incidence rates per 1,000 patient-years with 95% CI in parentheses.

Supplemental Table 2. Clinical Outcomes for Treatment of MI Subgroup Using Adjusted Models*

|  | Unadjusted | | Adjusted | | |  |
| --- | --- | --- | --- | --- | --- | --- |
|  | HR (95% CI) | *p*-value | HR (95% CI) | *p*-value | | |
| All-cause mortality | 0.63 (0.37 to 1.09) | 0.100 | 0.80 (0.42 to 1.51) | 0.493 |  |  |
| Myocardial infarction | 2.13 (1.32 to 3.46) | 0.002 | 2.47 (1.35 to 4.52) | 0.003 |  |  |
| Stent thrombosis | 5.05 (0.46 to 55.81) | 0.186 | ---- | ---- |  |  |
| Target lesion revascularization | 0.96 (0.34 to 2.71) | 0.948 | 1.31 (0.37 to 4.56) | 0.674 |  |  |
| CABG | ---- | ---- | ---- | ---- |  |  |
| Cardiac readmission | 0.90 (0.63 to 1.31) | 0.602 | 0.93 (0.60 to 1.45) | 0.761 |  |  |

*Adjusted model outcomes were adjusted for all baseline and procedural characteristics
